# High-Throughput SHAPE Analysis Reveals Structures in HIV-1 Genomic RNA Strongly Conserved across Distinct Biological States

**DOI:** 10.1371/journal.pbio.0060096

**Published:** 2008-04-29

**Authors:** Kevin A Wilkinson, Robert J Gorelick, Suzy M Vasa, Nicolas Guex, Alan Rein, David H Mathews, Morgan C Giddings, Kevin M Weeks

**Affiliations:** 1 Department of Chemistry, University of North Carolina, Chapel Hill, North Carolina, United States of America; 2 AIDS Vaccine Program, SAIC-Frederick, National Cancer Institute-Fredrick, Frederick, Maryland, United States of America; 3 Department of Biomedical Engineering, University of North Carolina, Chapel Hill, North Carolina, United States of America; 4 Swiss Institute of Bioinformatics, Lausanne, Switzerland; 5 HIV Drug Resistance Program, National Cancer Institute-Fredrick, Frederick, Maryland, United States of America; 6 Department of Biochemistry and Biophysics, University of Rochester Medical Center, Rochester, New York, United States of America; 7 Department of Microbiology and Immunology, University of North Carolina, Chapel Hill, North Carolina, United States of America; 8 Department of Computer Science, University of North Carolina, Chapel Hill, North Carolina, United States of America; Stanford University, United States of America

## Abstract

Replication and pathogenesis of the human immunodeficiency virus (HIV) is tightly linked to the structure of its RNA genome, but genome structure in infectious virions is poorly understood. We invent high-throughput SHAPE (selective 2′-hydroxyl acylation analyzed by primer extension) technology, which uses many of the same tools as DNA sequencing, to quantify RNA backbone flexibility at single-nucleotide resolution and from which robust structural information can be immediately derived. We analyze the structure of HIV-1 genomic RNA in four biologically instructive states, including the authentic viral genome inside native particles. Remarkably, given the large number of plausible local structures, the first 10% of the HIV-1 genome exists in a single, predominant conformation in all four states. We also discover that noncoding regions functioning in a regulatory role have significantly lower (*p*-value < 0.0001) SHAPE reactivities, and hence more structure, than do viral coding regions that function as the template for protein synthesis. By directly monitoring protein binding inside virions, we identify the RNA recognition motif for the viral nucleocapsid protein. Seven structurally homologous binding sites occur in a well-defined domain in the genome, consistent with a role in directing specific packaging of genomic RNA into nascent virions. In addition, we identify two distinct motifs that are targets for the duplex destabilizing activity of this same protein. The nucleocapsid protein destabilizes local HIV-1 RNA structure in ways likely to facilitate initial movement both of the retroviral reverse transcriptase from its tRNA primer and of the ribosome in coding regions. Each of the three nucleocapsid interaction motifs falls in a specific genome domain, indicating that local protein interactions can be organized by the long-range architecture of an RNA. High-throughput SHAPE reveals a comprehensive view of HIV-1 RNA genome structure, and further application of this technology will make possible newly informative analysis of any RNA in a cellular transcriptome.

## Author Summary

The function of the RNA genome of the human immunodeficiency virus (HIV) is determined both by its sequence and by its ability to fold back on itself to form specific higher-order structures. In order to describe physical structures in a region of the HIV RNA genome known to play multiple, critical roles in viral replication and pathogenesis, we invent a high-throughput, quantitative, and comprehensive structure-mapping approach that locates flexible (unpaired) nucleotides within a folded RNA, assaying hundreds of nucleotides at a time. We find that the first 10% of the HIV-1 genome has a single predominant structure and that regulatory motifs have significantly greater structure than do protein-coding segments. The HIV genome interacts with numerous proteins, including multiple copies of the nucleocapsid protein. We directly map RNA–protein interactions inside virions and discover that the nucleocapsid prottein interacts with viral RNA in at least three distinct ways, depending on the context within the overall genome structure. Further application of the high-throughput RNA-structure analysis tools described here will make it possible to address diverse structure–function relationships in intact cellular and viral RNAs.

## Abstract 3

Development of novel, quantitative, high-throughput RNA structure analysis tools allows the outline of structure-function relationships for the first 10% of an HIV genome, discovery of structural differences between regulatory and coding regions, and analysis of protein-RNA interactions inside authentic virions.

## Introduction

As is the case with many natural RNAs, the function of the HIV RNA genome is strongly linked to its ability to form higher-order structure and to interact with protein effectors in each stage of its replication cycle. The 5′ end of the HIV genome alone contains a noncoding, highly structured region that is involved in viral packaging, dimerization, pairing with the cellular tRNA primer for cDNA synthesis, and binding numerous viral proteins [1,2].

Immediately downstream of this regulatory region, the HIV genome contains nine open reading frames. The first protein encoded by the HIV-1 genome is Gag, which is ultimately cleaved into a complex set of proteins, including matrix, capsid, and nucleocapsid [2]. The nucleocapsid protein has two dissimilar RNA binding activities. First, as part of the Gag precursor protein, the nucleocapsid domain specifically recognizes and directs the HIV genome to be packaged into new virions [3–5] in the context of a vast excess of other cellular RNAs. In contrast, the nucleocapsid protein also has a significant duplex destabilizing activity, which plays a key role in facilitating the RNA annealing and rearrangement events essential for viral reverse transcription [6,7].

Because of its importance in regulating replication and for governing interactions with protein cofactors, extensive efforts have been directed towards developing structural models for the HIV-1 genome and for identifying candidate interaction sites for the nucleocapsid protein. Multiple models based on phylogenetic, chemical mapping, and mutagenesis approaches have been proposed [8–17]. In general, agreement between proposed structures is limited to highly structured hairpins; large zones of disagreement between models are common. The available experimental and sequence information is not sufficient to support one model over another. This lack of consensus in understanding HIV genome structure reflects challenges common to the analysis of any large RNA in its native biological context.

One of the most successful methods for determining an RNA secondary structure has been phylogenetic covariation analysis [18,19]. However, this method has a narrow range of usefulness in that homologous RNAs must be similar enough to form the same structure, but simultaneously exhibit sufficient polymorphism to be informative. Few RNAs meet this standard. In the case of HIV and most viral RNAs, sequences are typically too similar to each other to provide sufficient constraints over large regions.

Alternatively, RNA structural information can be inferred by treatment with chemicals or enzymes that discriminate between paired and unpaired nucleotides [20,21]. This reactivity information is then used to choose among various models, usually predicted with the assistance of thermodynamic folding algorithms. Conventional chemical and enzymatic mapping information has a narrow dynamic range and is usually available only for 25% to 50% of nucleotides in an RNA. As a result, conventional mapping experiments tend to focus on short pieces of RNA in artificial contexts and rely heavily on thermodynamic prediction and extrapolation to relate structures and protein binding sites identified in vitro to the biology of large RNAs in their native contexts. When used against intact viral particles, conventional probes have thus far also been unable to detect protein–RNA interactions inside HIV virions [16].

**Figure 1:**
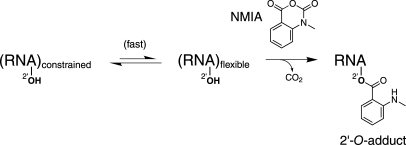
Scheme for RNA SHAPE Chemistry

Therefore, comprehensive and accurate analysis of any cellular or viral RNA in a relevant biological context requires a new technology that may be used for any RNA sequence under a wide variety of biologically relevant conditions. Our approach is based on the observation that electrophiles, such as N-methylisatoic anhydride (NMIA), react selectively with flexible RNA nucleotides at the ribose 2′-hydroxyl group (Figure 1) [22].

The RNA is exposed to NMIA at a concentration that yields approximately one 2′-*O*-adduct per 300 nucleotides (nts). Adducts are detected by their ability to inhibit primer extension by reverse transcriptase. A control extension reaction omitting NMIA to assess background, along with dideoxy sequencing extensions to assign nucleotide positions, are performed in parallel. These combined steps are called selective 2′-hydroxyl acylation analyzed by primer extension, or SHAPE [22–24]. Because the four canonical RNA nucleotides each contain a 2′-hydroxyl group, local nucleotide flexibility at all sites in an RNA is quantitatively interrogated in a single experiment.

In this work, we couple SHAPE chemistry with automated data readout and analysis systems so that hundreds of nucleotides of RNA structure can be analyzed in a single experiment. The overarching impact of this technology begins to realize the goal of making RNA structure analysis roughly as simple as contemporary DNA sequencing. High-throughput SHAPE (hSHAPE) makes it possible to rapidly measure RNA backbone flexibility at greater than 90% of the nucleotides in an RNA under a variety of biologically relevant conditions.

To demonstrate the power of hSHAPE while examining one of the most functionally important regions of an HIV genome, we applied hSHAPE to the first 900 nts of the 5′ end of the HIV-1 genome, under four biologically informative states. The data from these experiments allow us to address a comprehensive set of challenges that all require accurate knowledge of the structure of the HIV RNA genome: Does the viral genome have a global, long-range architecture, or is it largely organized as a series of isolated structural elements? Is it possible to identify RNA regulatory motifs based on their underlying RNA structure? Can consensus RNA binding sites be identified for the viral nucleocapsid protein, and if so, are these organized in domains? Can nucleocapsid interaction sites that function in specific RNA binding and genome packaging be distinguished from those that are targets for duplex destabilization by this same protein? If two classes of interaction sites for the nucleocapsid protein can be identified, do the specific binding and duplex destabilization activities operate in the same or in neighboring regions of the HIV genome?

## Results

### hSHAPE Strategy

**Figure 2:**
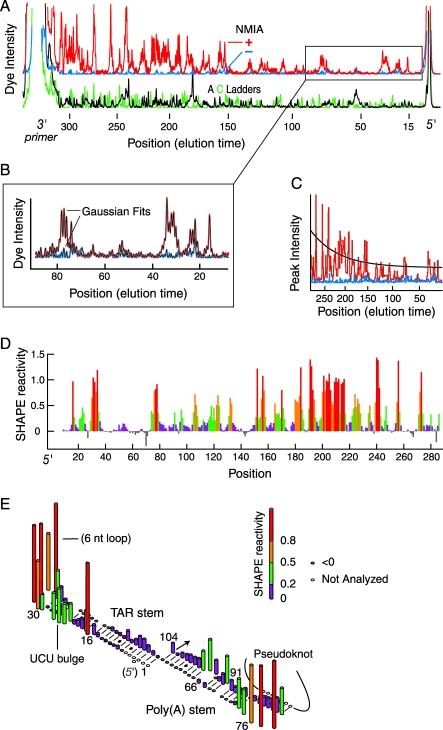
Analysis of HIV-1 RNA Genome Structure Using hSHAPE (A) Intensity versus elution time for an hSHAPE analysis resolved by single capillary electrophoresis using the HIV-1 in vitro transcript. The (+) and (−) NMIA reactions (red and blue) are offset from the A and C sequencing lanes (black and green) for clarity. (B) Whole-trace peak integration of a section of data from part (A). (+) and (−) peaks were fit to Gaussian curves (thin black lines) and aligned to the sequencing channels. (C) Signal decay correction for the (+) NMIA trace assuming a constant probability for extension at each nucleotide (black line). (D) Processed SHAPE reactivities as a function of nucleotide position. Highly reactive nucleotides (red and orange bars) report flexible positions in the RNA. (E) Absolute SHAPE reactivities superimposed on a secondary structure model for the TAR and Poly(A) stem-loops.

To create the technology necessary to analyze long regions of an RNA in biologically relevant environments in a single experiment, we performed each extension using a primer labeled with a color-coded fluorophore. The resulting cDNA products (from the (+) and (−) reagent reactions plus two sequencing traces) are combined and resolved in one multi-fluor run by automated capillary electrophoresis. In a single multiplex hSHAPE read, we typically obtain 250–400 nts of quantitative RNA structural information at single-nucleotide resolution (Figure 2A).

These elution time versus dye amount data resemble DNA sequencing information analyzed by capillary electrophoresis, and require similar preprocessing steps, such as baseline correction and corrections for fluorescent multiplexing [25]. However, whereas DNA sequencing requires detection of only the most intense peak at each position, the amplitudes in the (+) and (−) NMIA reagent channels in an hSHAPE experiment report quantitative RNA structural information. Peaks with little or no reactivity in the (+) NMIA channel correspond to RNA nucleotides constrained by base pairing or other interactions, whereas tall peaks indicate conformationally flexible positions (red and blue traces, Figure 2A). The dynamic range that distinguishes flexible from paired nucleotides is approximately 30-fold.

**Figure 3:**
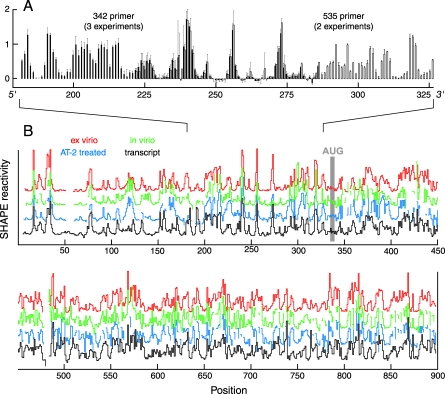
Single Nucleotide Resolution hSHAPE RNA Structure Analysis Is Quantitative and Highly Reproducible (A) SHAPE reactivities of two overlapping extension reactions for the in vitro transcript using primers that anneal approximately 200 nucleotides apart. Solid and open bars indicate normalized SHAPE reactivities for primers that anneal at nucleotides 342–362 and 535–555, respectively. Error bars represent standard deviations calculated from independent experiments using the same primer. (B) Complete SHAPE data for HIV-1 genomic RNA analyzed in virions and in the ex virio, AT-2–treated, and in vitro transcript states (see Dataset S1).

**Figure 4:**
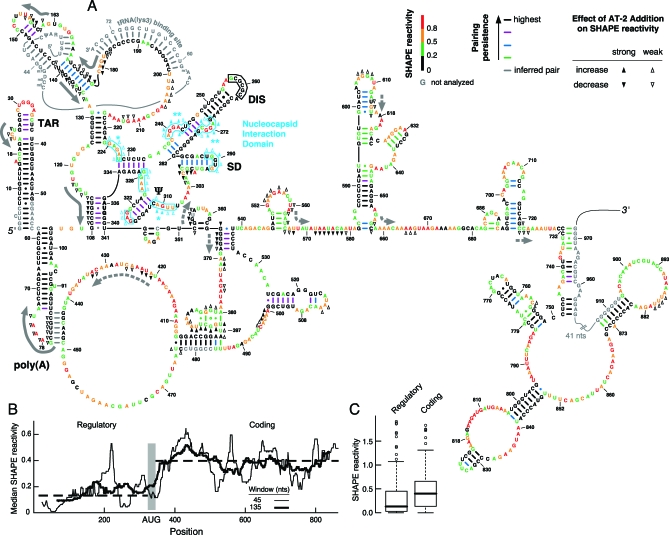
Architecture and Protein Modulation of the Structure of the HIV-1 NL4–3 RNA Genome (A) Secondary structure model generated by SHAPE-constrained folding of the ex virio state. All four states fold to this structure. Nucleotides proposed to form intermolecular base pairs with a second monomer are enclosed within a black box; the AUG start codon for the Gag polyprotein (nucleotide 336) is boldface. Nucleotides are colored according to their SHAPE reactivity; bars indicating base pairing are colored by their pairing persistence. Effects of pretreatment of viral particles with AT-2 are indicated with closed and open arrowheads. Clustered sites that show a strong increase in reactivity with AT-2 treatment (and report specific nucleocapsid binding sites) are emphasized in cyan; proposed primary and secondary sites are identified with double (**) and single asterisks (*), respectively. Sites showing a strong reduction in SHAPE reactivity (and reporting the structure destabilizing activity of nucleocapsid) are emphasized with solid and dashed gray arrows. The bound tRNA(lys3) is shown starting at nucleotide 33. The pseudoknot involving positions 75–84/443–449 [14] was not predicted directly by our algorithm, but by inference because both loop regions were unreactive towards SHAPE chemistry. (B) The 5′ regulatory domain is more highly structured than the 3′ coding region. Median SHAPE reactivities (solid lines) were calculated over rolling windows of 45 and 135 nts. Median reactivities for the entire 5′ regulatory and 3′ coding regions (dashed lines) are 0.13 and 0.40, respectively. (C) Box plot analysis [64] of distinct reactivity distributions for the 5′ and 3′ domains. Boxes outline middle 50% of each dataset; medians are shown with heavy lines. Open circles indicate values >1.5 times the interquartile range (boxed) and are considered outliers. The fences (small horizontal lines above and below the box) are the largest or smallest non-outlier values.

We calculated quantitative SHAPE reactivity information at every nucleotide position by developing hSHAPE-specific processing algorithms that (1) align the (+) and (−) NMIA channels to the RNA sequence (Figure 2A), (2) integrate the (+) and (−) NMIA peaks (Figure 2B), (3) correct signal decay (black line, Figure 2C), and (4) normalize SHAPE reactivities to a universal scale (Figure 2D). In this case, these steps produce a single-nucleotide resolution view of RNA flexibility for HIV-1 nucleotides 8 through 286 (Figure 2D).

To verify the accuracy of hSHAPE, we superimposed SHAPE reactivities on the well-characterized TAR and poly(A) stem-loops (nucleotides 1–104), which is the only region in the first 900 nts of the HIV RNA genome longer than approximately 50 nts for which prior analyses have converged on a single structural consensus [9,15,16]. The SHAPE reactivity information is exactly consistent with the consensus secondary structure model for this region (Figure 2E). Nucleotides with normalized SHAPE reactivities greater than 0.5 are almost always single stranded (orange and red columns, Figure 2E), whereas positions with reactivities less than approximately 0.2 (purple bars, Figure 2E) are almost always paired. Reactivities between these values (green) may be paired or participate in other partially constraining interactions. SHAPE reactivities also accurately report fine-scale structural differences. For example, nucleotides in the UCU bulge in the TAR stem show intermediate reactivities (Figure 2E), consistent with nuclear magnetic resonance (NMR) studies [26] that indicate that these nucleotides are partially stacked.

### Structural Analysis of Four HIV-1 Genome States

Our goal was to determine the structure of and derive biological inferences for the 5′ region of the HIV-1 RNA genome, as it exists inside wild-type viral particles. As will be shown below, hSHAPE provides an extraordinarily detailed and high-resolution view of the HIV-1 genome inside authentic virions that reflects multiple biologically important RNA–RNA and RNA–protein interactions. To identify and characterize virion-specific RNA conformational changes and RNA–protein interactions, we used hSHAPE to analyze the structures of four states in total. In addition to (1) genomic RNA inside virions (the in virio state), we compared the structure of the in virio state with three simpler states involving (2) authentic HIV-1 genomic RNA that had been gently, but completely, deproteinized and extracted from virions (ex virio), (3) genomic RNA inside virions, but in which nucleocapsid-RNA interactions were selectively disrupted in situ by treatment with Aldrithiol-2 (AT-2 treated, described in detail below), and (4) a 976-nt HIV-1 transcript generated in vitro in the absence of any viral component. We used the protein-free ex virio RNA as the reference state for comparison. This RNA state is strongly influenced by the authentic virion environment but simultaneously lacks the complex influence of bound proteins.

We constructed quantitative, single-nucleotide resolution profiles for the first 900 nts, or 10% of the HIV-1 genome, for each of these four states by combining the information from overlapping, and highly reproducible (Figure 3A) hSHAPE reads. Two to three independent repetitions were obtained for each primer; standard deviations are small, on the order of 0.1 normalized SHAPE unit or less. By combining the results of 32 individual hSHAPE experiments of approximately 300 nts each, we obtained structural information for 94% of all nucleotides in these four states plus analysis of three structural mutants, for a total analysis of 9,100 nts (Figures 3B and S1).

### Structural Model of the HIV-1 Genome

The nucleotide-resolution SHAPE reactivities for the large analyzed region of the HIV-1 genome represent an unprecedented amount of structural information with which to characterize RNA structure, protein binding sites, and conformational changes that differentiate our four genome states. However, comprehensive single-nucleotide resolution data do not, by themselves, yield a secondary structure model for an RNA.

We incorporate SHAPE reactivity information as an additional quasi-energetic constraint into an existing thermodynamic model [27,28] for RNA secondary structure prediction. Algorithms used to predict RNA structures from sequence show large increases in accuracy when experimental constraints are included in the prediction [28–30]. Ongoing work from our laboratories shows that using SHAPE information to constrain RNA secondary structure prediction has a dramatic impact on the accuracy of predicted structures. For example, prediction accuracy improves from 52% to 90% for the RNase P specificity domain [30] and from 38% to approximately 90% for the 1,542-nt Escherichia coli 16S rRNA (unpublished data). These predictions feature overall topologies that closely resemble the correct structure, with errors generally limited to local rearrangements at multi-helix junctions.

SHAPE reactivities provide model-free information about the extent of structure at each nucleotide. Therefore, in addition to proposing a complete secondary structure for our ex virio reference state (Figure 4A), we directly assessed the well determinedness of each helix in the secondary structure by increasing the relative weight of the SHAPE information in calculating low free-energy structures. We term this analysis the “pairing persistence” of each helix. Highly persistent helices (black and purple bars indicating base pairing in Figure 4A) form even when SHAPE reactivities were used to impose large pairing penalties for even slightly reactive nucleotides. Less persistent helices (blue and green bars, Figure 4A) form only when the SHAPE contributions to the constraints are decreased.

### The 5′ Regulatory Domain Is More Highly Structured Than Adjacent mRNA-Like Coding Sequences

The 5′ end of the HIV genome contains two functional regions whose boundary is the AUG start codon for the Gag coding sequence (nucleotides 336–338; in bold, Figure 4A). Positions 5′ of the AUG start codon form a 340-nt–long noncoding regulatory domain that plays multiple important roles in the viral replication cycle. In contrast, nucleotides 3′ of the start codon contain the Gag protein coding region, of which we have analyzed approximately 560 nts.

An important unresolved issue has been whether regulatory motifs might be identified from single RNA sequences. This is a challenging problem, and it is currently not possible to distinguish coding versus noncoding regions by thermodynamic secondary structure prediction alone [31,32]. hSHAPE analysis directly addresses this issue in two ways.

First, the median SHAPE reactivity, a metric for the typical amount of structure in the two regions, is 0.14 for the 5′ regulatory domain and 0.40 for the 3′ mRNA-like region (dashed lines, Figure 4B). Differences in SHAPE reactivities between the two regions are statistically significant (Wilcoxon rank sum test *p*-value < 0.0001) with both the median and overall distribution being lower in the 5′ regulatory domain (Figure 4C). The inflection point in SHAPE reactivities is nearly coincident with the AUG start codon (gray bar, Figure 4B). Second, the predicted secondary structure model can be used to infer the density of stable base pairing interactions in the 5′ regulatory versus 3′ coding regions. Nucleotides in the 5′ regulatory domain are 1.7 times more likely to be paired than those in the 3′ coding region (Figure 4A). Although there are some flexible regions in the 5′ regulatory domain and a few stable duplexes in the 3′ coding region, overall there is a strong and statistically significant difference in the amount of structure in these two regions. Thus, by both criteria, hSHAPE clearly distinguishes between regulatory and coding regions within the HIV-1 genome because the noncoding regulatory domain is more highly structured than are coding sequences.

### The HIV-1 Genome Forms a Single Predominant Structure

Comparison of the complete SHAPE reactivity profiles for the ex virio reference state with three other very different states—in virio, in virio with compromised nucleocapsid–RNA interactions, and an in vitro transcript—unexpectedly reveals that these four distinct states contain extensive regions with essentially identical structures (Figure 3B). This is a remarkable result, considering the different biochemical histories of the four states. At the two extremes, the in virio RNA was maintained in its native conformation inside virions throughout the chemical interrogation step, whereas the in vitro transcript RNA did not interact with any authentic viral component, but instead, was synthesized and refolded entirely in vitro.

We performed several control experiments to test how well hSHAPE detects significant, but local, differences in RNA conformation. These experiments encompassed analyzing an in vitro transcript containing a deletion of the U^22^CU^24^ bulge in TAR, comparing the 976-nt in vitro transcript with a 3′ deletion that disrupts the pseudoknot at positions 79–85/443–449, and dissociating tRNA(lys3) from the ex virio RNA by a heating step. In each case, we detected strong changes in SHAPE reactivity exactly in the region whose structure was disrupted (see Figure S1). These experiments are consistent with prior work that demonstrates that SHAPE is exquisitely sensitive to small changes in local RNA structure [23,29,30,33–36] and indicates that if structural differences exist between any two of our HIV-1 states, they are detectable by hSHAPE.

In all normal retroviral particles, the genomic RNA exists as a dimer, with similar or identical RNA strands linked together by a limited number of base pairs and tertiary interactions. The dimeric structure appears to be a critical element in the selective packaging of the genome [37,38] and in template switching between two RNA monomers during reverse transcription that leads to recombination [39–41], a major source of genetic variation for retroviruses. Dimerization is a complex process that may involve multiple stem-loops in the genome, but no single motif has been shown to be absolutely necessary for dimerization.

**Figure 5:**
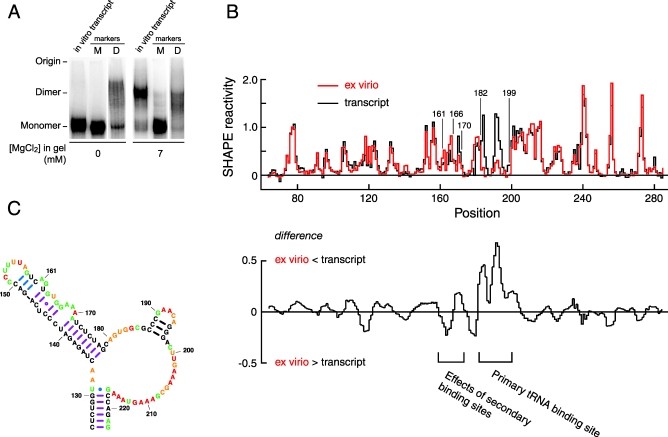
RNA Conformational Changes That Differentiate Ex Virio and In Vitro Transcript States (A) The in vitro transcript forms a loop-loop dimer. Identical samples of the transcript RNA were resolved in nondenaturing gels, either omitting or containing 7 mM MgCl_2_. Markers for monomeric (M) and dimeric (D) conformations are shown. (B) SHAPE reactivity as a function of nucleotide position for ex virio (red) and transcript (black) RNAs. Difference plot in lower panel has been smoothed over a 5-nt window. (C) Secondary structure model for the primer binding site in the absence of tRNA(lys3).

In order to assess the dimeric nature of our in vitro transcript, we resolved monomeric and dimeric conformations of this RNA in nondenaturing gels (Figure 5A). When the gel does not contain MgCl_2_, the HIV transcript RNA has a mobility identical to a monomer marker. In contrast, when Mg^2+^ is added to the gel and running buffer, the transcript runs as a well-defined species consistent with formation of a dimer. This is exactly the behavior expected if the in vitro transcript forms one or more Mg^2+^-dependent loop-loop interactions. A similar loop-loop dimer state has been identified for the Moloney murine sarcoma retrovirus [33,42].

Dimerization is thought to involve an initial loop-loop interaction [43] at the self-complementary sequence G^257^CGCGC^262^. We find that these nucleotides are unreactive in both the in vitro transcript and ex virio states (boxed nucleotides, Figure 4A). Thus, both SHAPE reactivities and the Mg^2+^-dependent behavior of the in vitro transcript strongly support formation of intermolecular base pairs at this loop.

In contrast, the genomic RNA obtained from HIV viral particles forms a dimer that is stable even when subjected to gel electrophoresis in the absence of Mg^2+^ [37]. We therefore sought to identify the structural differences that distinguish the loop-loop dimer formed by the in vitro transcript and the stable dimer in the ex virio state. However, we were unable to detect any statistically significant reactivity differences between the in vitro transcript and ex virio RNAs, including in sequences flanking the 257–262 loop (compare red and black traces in Figures 3B and 5B) or in base-paired regions of the TAR stem.

Nucleotide resolution SHAPE data are therefore inconsistent with models derived from in vitro experiments that postulate [40,43] that dimerization in mature viruses is mediated by the formation of stable intermolecular duplexes involving the stem sequences adjacent to the DIS and TAR loops. However, our analysis based on HIV-1 genomic RNA purified from virions is consistent with recent in vivo experiments that show neither the TAR stem [44] nor the ability to form intermolecular base pairs in the DIS stem [45] are required for viral replication. Instead, we suggest that HIV-1 genome dimerization is mediated by loop-loop interactions alone, potentially augmented by interactions that have not yet been identified. Another possibility is that large-scale structural changes occur, but yield almost identical local nucleotide flexibilities in the pre- and post-dimer RNAs. We think this is a remote possibility, given the ability of hSHAPE to detect small changes in local RNA structure (Figure S1).

Finally, within the predominant state for this large region of the HIV-1 genome, there are numerous regions that are persistently accessible to SHAPE chemistry. These regions are expected to hybridize readily with complementary sequences, including antisense and RNA interference (RNAi)-based oligomers, and represent multiple new and attractive targets for anti-HIV therapeutics (red and orange positions, Figure 4A).

### The tRNA(lys3) Binding Site

Reactivity profiles for the four states analyzed in this work do show limited structural differences, which are consistent with important, but local, RNA conformation and protein binding effects. Because hSHAPE gives quantitative information at each nucleotide position, structural differences are readily detected both by comparing two reactivity profiles or by subtracting one profile from another to yield a difference plot (for example, see lower panel in Figure 5B).

Over the 900 nts of analyzed sequence, there is a single region that shows significant differences between the ex virio reference state and the transcript RNA, refolded in vitro. This region lies between nucleotides 160 and 200 (Figure 5B). The most dramatic difference is that the ex virio state is much less reactive at positions 182 to 199 (Figure 5B). This region maps exactly to the main tRNA(lys3) primer binding site [1] and indicates that the primer is tightly paired to the HIV-1 RNA genome in viral particles. The in vitro transcript, which is not bound by tRNA, folds into a different local structure in these regions (compare the tRNA(lys3) binding sites in Figures 4A and 5C). We confirmed that these structural differences reflect tRNA binding by dissociating the tRNA from the genomic RNA via a heating step (Figure S1).

The tRNA primer also has the ability to form additional interactions with the genomic RNA, although current proposals for these interactions diverge widely [16,46,47]. The SHAPE data show a clear pattern in which the ex virio state is more reactive at positions 161–166 and less reactive at positions 168–170, as compared with the in vitro transcript. These reactivity changes are consistent with tRNA(lys3)-induced structural rearrangement in RNA sequences outside the primary 18-bp tRNA primer binding site. The SHAPE reactivities thus strongly support a model in which the cellular tRNA(lys3) molecule interacts with the HIV-1 genome via three distinct base-pairing interactions (Figure 4A, see tRNA(lys3) label).

### Direct Analysis of Nucleocapsid Protein–RNA Genome Interactions

**Figure 6:**
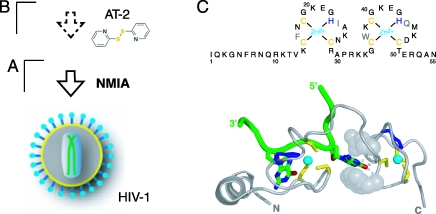
Analysis of the Effects of Nucleocapsid Binding on HIV-1 RNA Genome Structure by In Situ Disruption Using the Zinc-Ejecting Agent AT-2 (A and B) Schemes for analyzing HIV-1 genomic RNA structure in virio (A) and following zinc-ejection with AT-2 (B). (C) Amino acid sequence and an RNA-bound conformation [58] of the HIV-1 nucleocapsid protein. Nucleocapsid is gray, and the RNA strand is green. Bound Zn^2+^ is cyan; zinc ligands are yellow and blue. Conserved stacking interactions for the C-terminal zinc knuckle are shown by space-filling atoms; hydrogen bonds are shown as small spheres.

**Figure 7:**
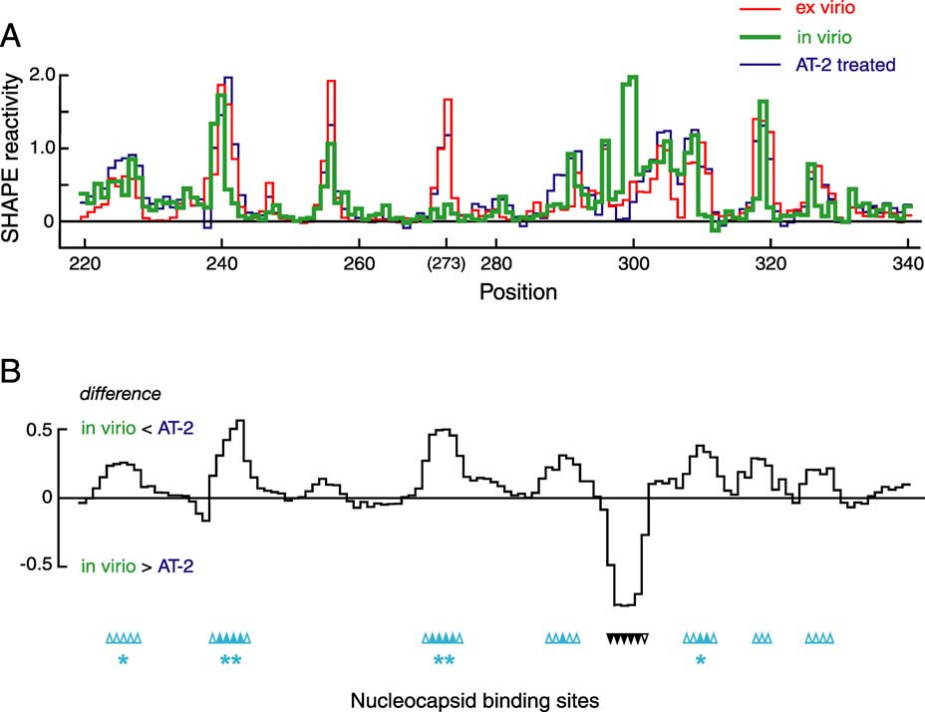
A Specific Nucleocapsid Binding Domain in the HIV-1 Genome (A) SHAPE reactivities as a function of nucleotide position for ex virio (red), in virio (green), and AT-2-treated (blue) states. (B) Difference plot illustrating the effect of AT-2 treatment on SHAPE reactivity relative to in virio genomic RNA. Sites of enhanced SHAPE reactivity upon compromising nucleocapsid zinc knuckle structures by AT-2 treatment are reported as positive peaks. Individual nucleotides showing large changes in reactivity are indicated with upward-pointing arrowheads and correspond to the same symbols shown in Figure 4A. Primary and secondary sites are emphasized with double (**) and single asterisks (*) , respectively. Reactivity differences are smoothed over a 5-nt window.

We analyzed the structure of HIV-1 genomic RNA inside native virions by treating intact viral particles with NMIA and then extracting and processing the modified RNA (the in virio state; see scheme in Figure 6A). Over large regions of the HIV-1 genome, SHAPE reactivity patterns for the in virio and protein-free ex virio states are very similar. These data emphasize that NMIA readily penetrates the viral membrane and yields quantitative information about HIV RNA structure in the context of the complex virion environment. We also observe reproducible local differences between the ex virio and in virio states that typically span 4–6 nts (compare red and green profiles, Figure 7A). These differences could reflect either different local RNA conformations, like those identified in the tRNA binding region (Figures 4A and 5C), or the effects of binding by viral proteins.

The most prominent protein ligand for genomic RNA in mature HIV virions is the nucleocapsid protein [3,6]. The nucleocapsid protein functions in two, almost diametrically opposed, ways. As part of the Gag precursor, the nucleocapsid domain binds specifically to the HIV genome to direct packaging of this RNA into nascent virions. As the mature nucleocapsid, this protein is thought to interact nonspecifically with RNA and thereby destabilize RNA duplexes and facilitate strand rearrangement and annealing events.

These activities are mediated, in part, by two copies of a CX_2_CX_4_HX_4_C motif that coordinate a zinc ion to form a compact “zinc knuckle” domain (Figure 6C). This domain has been shown to bind guanosine in model systems [7]. Thus far, it had not been possible to convincingly identify specific sites in the genome that bind or are destabilized by nucleocapsid or Gag proteins in virions.

In this work, we took advantage of the prior discovery that “zinc ejecting” agents like 2,2′-dithioldipyridine (or Aldrithiol-2 [AT-2]; Figure 6B) covalently modify cysteine residues in the zinc knuckle motifs and disrupt interactions between the zinc ion and its cysteine ligands. Treatment of viral particles with AT-2 promotes formation of reagent-nucleocapsid and nucleocapsid–nucleocapsid crosslinking and efficiently compromises nucleocapsid–RNA interactions [48–50]. AT-2–treated virions retain the ability to bind to target cells and to undergo membrane fusion as well as native viral particles; however, these virions are not infectious and cannot undergo the first steps of reverse transcription [49,50]. Thus, treatment with AT-2 severely compromises the RNA-binding activity of the nucleocapsid protein, but leaves the surface of the virus particle intact [49,50]. To detect nucleocapsid–RNA interactions inside intact viral particles, we therefore treated virions with AT-2 and then analyzed the structure of the resulting genomic RNA using hSHAPE (see scheme, Figure 6B). This approach constitutes a “reverse footprinting” experiment in which the effects of nucleocapsid–RNA interactions are detected by disrupting the zinc knuckle-RNA interactions inside virions.

### Nucleocapsid and HIV-1 Genome Structure

**Figure 8:**
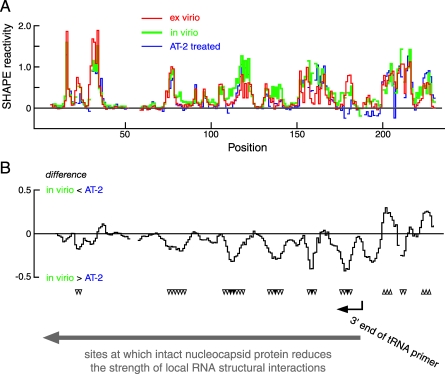
Nucleocapsid Increases SHAPE Reactivity and, by Inference, RNA Flexibility at Multiple Sites 5′ to the tRNA(lys3) Primer Binding Site (A) Absolute SHAPE reactivities for the ex virio (red), in virio (green) and AT-2 treated (blue) states. (B) Difference plot reporting the effect of AT-2 treatment on the in virio state. Position of bound 3′ end of the tRNA primer is shown with a black arrow; large gray arrow and downward-pointing arrowheads indicate sites of structure destabilization by nucleocapsid.

Three initial conclusions are apparent from comparison of the in virio with the ex virio and AT-2-treated states. First, regions showing strong changes in SHAPE reactivity in the AT-2-treated state almost always resemble the protein-free ex virio state (compare blue and red traces, Figures 7A and 8A). These experiments confirm that AT-2 treatment does disrupt nucleocapsid–RNA interactions inside virions, but importantly, that compromising the zinc knuckle structures in the nucleocapsid protein does not cause formation of nonspecific aggregating interactions with the genomic RNA.

Second, disrupting nucleocapsid–RNA interactions by AT-2 treatment both increases (Figure 7) and decreases (Figure 8) local nucleotide flexibility in distinct genome regions. The strongest and most densely arrayed effects of AT-2 treatment lie in the 5′ regulatory domain (upward and downward pointing arrowheads, respectively; Figure 4A). Thus, even in the context of an intact HIV-1 genome, nucleocapsid proteins recognize smaller motifs within this large RNA.

Third, we observe three different interaction motifs between nucleocapsid protein and the HIV-1 genome. In order to systematically locate and characterize these motifs, we subtract the SHAPE reactivities of the in virio state from the AT-2–treated state, and plot the smoothed differences (Figures 7B and 8B). These difference plots reveal locations in the RNA that are reproducibly affected by nucleocapsid protein binding.

Eleven sites show statistically significant (*p* < 0.001) increases in SHAPE reactivity in the AT-2–treated state. Seven of these lie in a single compact domain involving positions 224–334 in the genome (cyan, Figure 4A). The strongest single effect of compromising nucleocapsid–RNA interactions occurs at positions 271–274, followed closely by positions 239–244 (emphasized with double asterisks and upwards pointing arrowheads in Figures 4A and 7B). These sites, which have not been previously implicated in nucleocapsid or Gag recognition, are likely to be the primary interaction motifs for the viral nucleocapsid protein domain at the 5′ end of the HIV-1 genome. We also identify other sites with smaller AT-2–induced reactivity changes, which we characterize as secondary (single asterisks; positions 224–227 and 308–312, Figure 4A) and tertiary sites (positions 289–292, 318–320, and 326–329). All seven sites feature a general structural consensus characterized by a G-rich single-stranded sequence flanked by stable helices.

The remaining four sites that are not clustered in the nucleocapsid interaction domain also occur at G-rich single-stranded regions adjacent to a stable helix (positions 201–205, 381–398, 553–558, and 606–609). In sum, these experiments indicate that hSHAPE directly identifies RNA interaction motifs inside native virions, that nucleocapsid protein binds at specific sites, and that many nucleocapsid-binding motifs are clustered in a domain within the 5′ regulatory region of the HIV-1 genome.

### Structure Destabilizing Activity of Nucleocapsid

In addition to recognizing specific motifs in the HIV-1 genome, the nucleocapsid protein also functions to destabilize short RNA helices [6,7]. If the nucleocapsid protein were functioning as a duplex destabilizer inside virions, compromising the activity of nucleocapsid (by AT-2 treatment) would reduce local nucleotide flexibility, and thus decrease SHAPE reactivities, at defined sites.

Nucleocapsid-mediated destabilization of HIV RNA genome structure is thus reported as negative peaks in a difference analysis (Figure 8B). Comparison of SHAPE reactivities for the in virio state with those for the AT-2 state in a difference analysis indicates that there are two statistically significant (*p* < 0.001) classes of sites in which local RNA structure is destabilized by intact nucleocapsid protein. A series of very strong effects is seen over the first 185 nts of the genomic RNA (emphasized with solid gray arrows, Figures 4A and 8B). No other region in the first 900 nts of the HIV-1 genome shows this concentrated and large-magnitude structural destabilizing activity by nucleocapsid. We also observe additional sites that lie at relatively widely spaced intervals in the coding region that experience enhanced SHAPE reactivity upon compromising the zinc knuckle motif (dashed gray arrows, Figure 4A). Thus, these experiments emphasize the unanticipated result that the helix-destabilizing activity of the nucleocapsid protein does not fall randomly throughout the genomic RNA, but instead, has its greatest effect in a compact domain immediately 5′ to the tRNA binding site.

## Discussion

### A Structure for the HIV-1 Genome

The first 10% of the HIV-1 RNA genome represents a microcosm of many possible classes of interactions that occur in any large cellular RNA. This RNA contains an extensive noncoding regulatory domain and also serves as a template for protein synthesis. The RNA also contains large-scale structures that are important for forming a dimeric state that facilitates genetic recombination, for base pairing with a specific cellular tRNA, and for binding of the viral Gag protein or nucleocapsid domain. A complete understanding of HIV RNA genome structure holds promise for identifying new targets for anti-HIV interventions.

**Figure 9:**
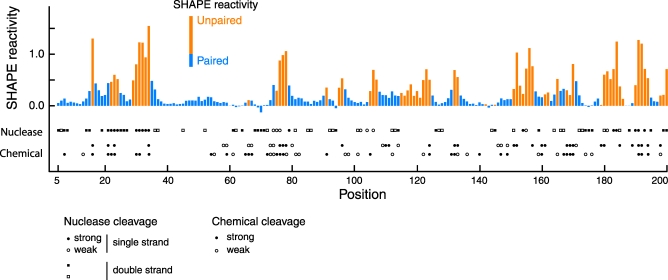
Density of SHAPE Reactivity Information Compared with Analyses of Related HIV-1 Sequences Using Conventional Chemical and Enzymatic Probes The histogram illustrates SHAPE reactivity as a function of position for the in vitro transcript RNA. Bars are colored according to whether the nucleotide is predicted to be paired (blue) or single stranded (orange). The results of nuclease mapping [15] (HXB2 isolate) and chemical mapping studies [8,16] (top, HXB2; bottom, MAL) are indicated below the SHAPE histogram. All mapping data were aligned with the HIV-1 NL4–3 sequence. This plot shows the most information-dense genome regions as analyzed by conventional approaches; significantly less information was available 3′ of position 360, and none was available 3′ of position 720 in the first 10% of the HIV-1 genome.

A critical attribute of hSHAPE analysis is that it is both more complete and quantitative than conventional approaches. This difference is readily seen by comparing the single-nucleotide resolution SHAPE information with prior analyses of HIV genome structure (Figure 9). It is clear that all of these studies are mapping nearly identical RNA conformations. Nucleotides reported to be reactive towards single-strand selective enzymatic [15] or chemical [8,16] reagents almost always have high SHAPE reactivities; whereas, nucleotides that are cleaved by V1 nuclease, which shows a preference for double-stranded RNA, exhibit low SHAPE reactivity.

Our secondary structure model is most similar to the proposal of Damgaard et al. [15], but still contains many substantive differences with respect to this and other models that reflect three innovations unique to the hSHAPE approach. First, hSHAPE yielded structural information for 94% of all nucleotides in the analyzed region, which is far more than any prior effort. Near completeness is essential because RNA secondary structure prediction in the absence of constraining reactivity data yields structures with significant errors, especially as RNA length increases [51–53]. In the case of HIV-1 genomic RNA, relatively little data had been obtained for positions 110–145, 220–243, 276–282, 370–450, and 465–530, and no data were available 3′ of position 720. Second, our model reduces the influence of end-effect artifacts that occur with analysis of small RNA fragments because it is generated via hSHAPE using full-length genomic RNA. For example, structures that involve or lie inside of long-range interactions, such as the 108–114/335–341 stem (called the U5-AUG interaction [15]) are mispredicted if the RNA sequence does not include the complete domain. Third, incorporation of SHAPE reactivity information as a pseudo-free energy change term makes the structure prediction calculation relatively insensitive to errors in any single reactivity measurement.

hSHAPE analysis of four biologically relevant states of the HIV-1 genome indicates that this RNA has a single, strongly conserved structure. This result challenges previous proposals for multiple conformations in this region of the genome [13,40,43] and likely reflects that we maintained the native conformation of authentic, long HIV-1 RNAs. Additional conformations may exist at other stages of the viral infectivity cycle.

hSHAPE analysis also indicates that regulatory and protein coding regions in HIV-1 are structurally distinct as judged by their quantitative SHAPE reactivities. Regulatory domains are more highly structured than coding sequences (Figure 4B and 4C). hSHAPE may be a broadly useful tool for identifying highly structured regulatory motifs in other viral and cellular RNAs.

### Three Classes of Sites at Which Nucleocapsid Modulates HIV Genome Structure

Understanding the RNA binding specificity and functions of the HIV nucleocapsid protein has proven to be challenging both because nucleocapsid has opposing specific and nonspecific binding activities and also because preferred RNA binding sites had not been clearly defined. In addition, previous in vitro mapping experiments using dimethyl sulfate failed to identify any HIV RNA–protein interactions [16], likely because the limited structural sensitivity of this reagent. To overcome these challenges, we invented experiments that focused on the native RNA binding activity of the nucleocapsid protein inside wild-type virions.

We used the zinc ejecting agent AT-2 [50] (Figure 6) to compromise nucleocapsid–RNA interactions inside virions. The effects of disrupting nucleocapsid–RNA interactions by AT-2 treatment are highly specific because changes in SHAPE reactivity are always localized to a small set of continuous nucleotides at each site. Notably, we observe structural changes consistent with both the specific RNA binding and with the duplex destabilizing activities of nucleocapsid.

As judged by (1) conserved sequences and structures and (2) their location in the genome, we identify three classes of nucleocapsid protein interactions with the HIV-1 genome. For all three classes, measured differences between the in virio and AT-2–treated states were highly statistically significant (*p* < 0.001), whereas, reactivity differences between nucleotides outside of these sites were statistically equivalent. The first class is a specific nucleocapsid binding site motif, and the other two classes are sites at which nucleocapsid destabilizes RNA duplex structure.

#### Class 1: Specific nucleocapsid binding motif.

**Figure 10:**
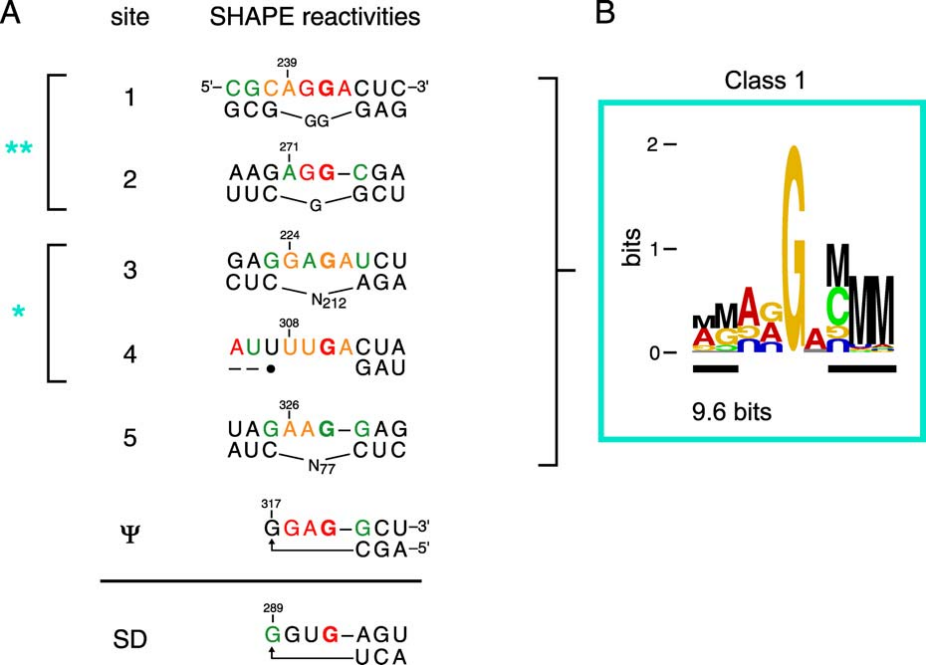
Nucleocapsid Binding Sites in the 5′ Regulatory Domain and Their Sequence and Structural Consensus Sites shown all exhibit statistically significant (*p* < 0.001) increases in SHAPE reactivity upon compromising RNA-nucleocapsid interactions by AT-2 treatment. (A) Alignment of internal loop (sites 1–5) and stem-loop (Ψ and SD) nucleocapsid binding sites (from Figure 4A). Nucleotides are colored by their SHAPE reactivity: red and orange indicate highly reactive positions; black nucleotides are unreactive. Primary and secondary sites are emphasized with double (**) and single (*) asterisks. N indicates the number of intervening nucleotides on the lower strand. (B) Information content for sites 1–5. Duplex regions are identified by black bars. Height of each letter indicates information. M represents mutual information from base pairing [54,55]; each nucleotide is color coded by base identity. For clarity, only the top strand is shown.

Between positions 224 and 328 there are seven sites at which AT-2 treatment strongly increased nucleotide flexibility. Sites 1 through 5 are single-stranded internal loop motifs flanked by double-stranded regions, whereas the remaining two sites (called the Ψ and SD elements) form short, stable RNA hairpins (Figure 10A). These interaction motifs can be characterized both structurally, using SHAPE reactivities, and also by their aggregate information content.

Sites 1–5 and Ψ within the nucleocapsid interaction domain share a consensus motif comprised of a purine-rich flexible region of 3–4 nts adjacent to a helix that usually terminates in a C-G base pair. Identification of this structural consensus was only possible in the context of the new, SHAPE-constrained, secondary structure. These sites share both a limited sequence homology and a characteristic experimental SHAPE reactivity profile (above the solid line, Figure 10A). Analysis of the information content in these sites indicates that the most prominent determinants for specific RNA binding are a single-stranded guanosine residue followed by a 3′ double-stranded region. The total information content [54,55] of this site is 9.6 bits (Figure 10B). The single-stranded guanosine is an important feature of the binding site, consistent with structural and biochemical analyses of nucleocapsid–RNA interactions [56–59]. However, this nucleotide represents only one-fifth of the total information in the site, which emphasizes that the overall structural context plays an equally important role in defining a nucleocapsid protein binding site.

The seventh site, the splice-donor (SD) stem-loop, is homologous in terms of sequence information; however, the flexible loop does not conform with the reactivity consensus because the loop nucleotides are unreactive towards SHAPE (see black nucleotides in the SD site, Figure 10A). NMR experiments show that nucleocapsid interacts with the Ψ and SD hairpins in completely different ways, involving protein interactions at opposite faces of an RNA helix [58,59], which reinforces the SHAPE analysis that local structure and nucleocapsid protein binding at these two sites are distinct.

The six sites that conform to both the information content and the SHAPE structure consensus (above the solid line, Figure 10A) lie in a single domain in our structure model for the 5′ end of the HIV-1 genome. This nucleocapsid recognition domain overlaps the packaging signal [3] and the region that forms a loop-loop interaction in the genomic RNA dimer (Figures 4A and 5A). Our experiments support a model in which the 223–334 domain dimer interacts with multiple nucleocapsid molecules and potentially with the nucleocapsid motif in Gag proteins. The specific juxtaposition of multiple high-affinity nucleocapsid/Gag binding sites in the dimer would then function as the structural motif that specifically directs HIV genomic RNA to be packaged in nascent HIV virions.

#### Classes 2 and 3: Nucleocapsid-mediated duplex destabilization.

**Figure 11:**
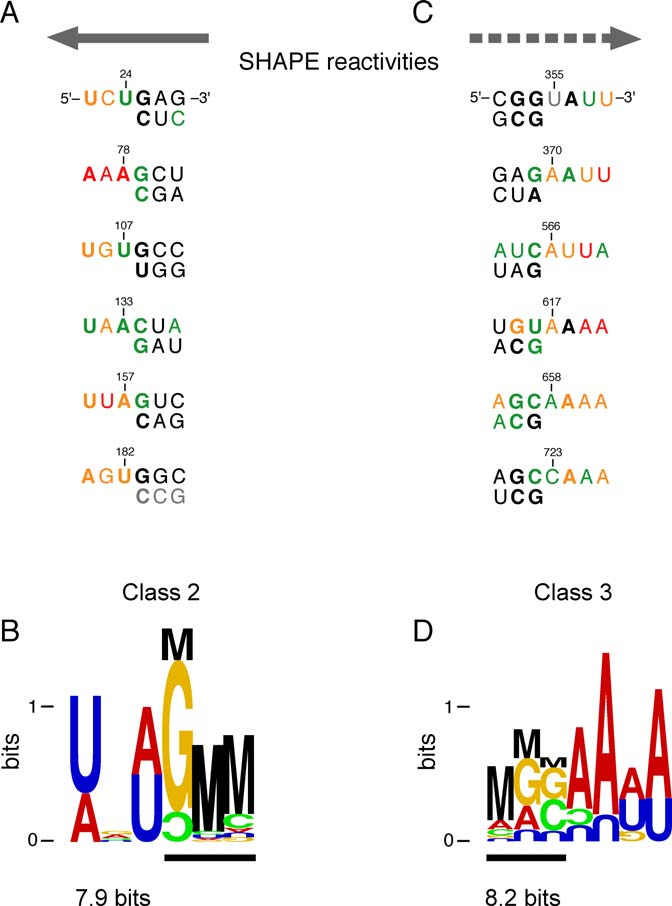
Alignment and Information Content at Sites where AT-2 Treatment Decreases SHAPE Reactivity (A) Alignment and (B) information content of the six Class 2 sites in the 5′ regulatory domain (gray arrow as in Figure 4A). Symbols are described in Figure 10. (C) Alignment and (D) information content of six Class 3 sites in the 3′ coding region (dashed arrow as in Figure 4A).

We also detect two classes of conserved motifs at which intact nucleocapsid protein increases local flexibility. In Class 2 sites, nucleocapsid destabilizes RNA structure at six compact regions between the 5′ end of the genome and the tRNA primer binding site (Figure 8B; and solid gray arrows, Figure 4A). These sites share both a consensus structure and significant sequence similarities (Figure 11A). The Class 2 motif consists of an A/U-rich single-stranded element followed by a duplex region initiated by a G-C base pair (with the G usually on “top”; Figure 11B). For all sites except the one containing position 107, the largest differences in SHAPE reactivity are asymmetrically distributed across the consensus motif. Nucleocapsid increases local nucleotide flexibility in the single-stranded region and at 1–2 nts in the “top” duplex strand (compare Figures 11A and 4A).

The six sites in the 3′ coding region, termed Class 3 sites, conform to a different consensus in which nucleocapsid protein destabilizes RNA structure (dashed arrows, Figure 4A). These six sites can also be characterized by their SHAPE reactivities and by the net information content of their sequences. Strikingly, these motifs share a consensus that is different from the duplex destabilized sites in the 5′ regulatory domain (Figure 11C). The motif contains a 5′ duplex that terminates in a G-C or other G-containing base pair followed by an A/U-rich single-stranded region (Figure 11D). For Class 3 motifs, AT-2–mediated reductions in SHAPE reactivities generally span the “top” strand of the duplex and then extend in a 3′ direction into the single-stranded region.

Class 2 and 3 sites share important features: both include single-stranded A/U-rich motifs adjacent to a duplex in which the first base pair includes a guanosine nucleotide. We propose that the duplex destabilizing activity of the nucleocapsid protein reflects protein binding to these guanosine residues and binding-induced destabilization of the rest of these short duplexes. The motifs differ in that the single-stranded elements lie 5′ versus 3′ of the duplex. We hypothesize that this asymmetry is linked to the function of nucleocapsid in facilitating distinct steps in the retroviral replication cycle. Class 2 sites all lie immediately 5′ to the tRNA(lys3) binding site and therefore represent the first nucleotides traversed by the viral reverse transcriptase during viral DNA synthesis (solid gray arrows, Figure 4A). These data are consistent with a model in which nucleocapsid facilitates the initiation of reverse transcription of the viral genome. In contrast, nucleocapsid interactions at Class 3 sites may have evolved to promote a different process involving HIV RNA, like facilitating translation of the 3′ coding sequences and movement of the ribosome during translation.

### Perspective

Using hSHAPE technology, we measured structure at 94% of the first 900 nts of the HIV-1 genome under seven sets of instructive conditions for a total analysis of 9,100 nts. Comparing structural information from these states indicates that large regions of the HIV-1 genome form a single structure, that regulatory motifs in the genome are highly base paired, that specific and nonspecific RNA binding modes of the nucleocapsid protein are manifest via interactions with domain-like elements of the RNA, and that the duplex destabilizing activity of nucleocapsid is mediated by two distinct classes of RNA sites. Because of its completeness, hSHAPE analysis provides information sufficient to discriminate between otherwise contradictory models for the HIV-1 genome.

Just as DNA sequencing has revolutionized our understanding of DNA genome function, high-throughput RNA structure analysis makes possible investigation of intact RNAs from any viral or cellular transcriptome, as a function of multiple biological states. We anticipate that hSHAPE will contribute significantly to establishing the connections between RNA structure and translational regulation, alternative splicing, binding by small interfering and related small RNAs, higher-order folding and packaging in viruses, and many other RNA-based processes.

## Materials and Methods

### HIV-1 particle production.

VSV-G pseudotyped HIV-1 NL4–3 viral particles were produced by cotransfecting the pNL4–3 and pHCMV-G (VSV-G protein expression construct) [60] plasmids at a ratio of 3:1 into 293T cells as described [61], except that TransIT293 (Mirus Bio) was used as the transfection agent. In sum, 40 × 150-cm^2^ culture flasks, seeded at a density of 3 × 10^6^ 293T cells, were transfected with plasmids 72 h later. Cultures were then incubated for 48 h and supernatants harvested, clarified by centrifugation at 5,000 *g* for 10 min, filtered through a 0.2-μm membrane, and stored at 4 °C overnight. Cultures were incubated for an additional 24 h with fresh culture medium, and virus-containing supernatant was again collected using the same procedure. Supernatants from both harvests were pooled and incubated at 4 °C in preparation for treatment with the AT-2 and NMIA reagents. Viral genomes were quantified by real-time reverse-transcription PCR (RT-PCR) [61]; the yield is typically 20 pmol HIV-1 RNA genomes/l cell culture.

### HIV-1 particle treatment with AT-2.

Aldrithiol-2 (AT-2, systematic name 2,2′-dithiodipyridine; 0.5 M in DMSO, 2.0 ml) or DMSO (2.0 ml) was added to 1.0 l of virus-containing supernatant and incubated overnight at 4 °C. Virus particles from the (+) and (−) AT-2 experiments were pelleted separately by centrifugation (110,000 *g*, 4 °C, 1.5 h) through a 20% (w/v) sucrose cushion in phosphate buffered saline. Pellets from 0.5 l of culture fluid were resuspended in 1.0 ml of NMIA reaction buffer (50 mM Hepes [pH 8], 200 mM NaCl, 0.1 mM EDTA, and 10% fetal bovine serum).

### NMIA modification of viral particles.

Concentrated samples of either purified viral particles or particles treated with AT-2 (500 μl) in NMIA reaction buffer were treated with NMIA (50 μl, 100 mM in DMSO) or neat DMSO (50 μl) for 50 min at 37 °C. The virus particle production, AT-2 treatment, and NMIA modification steps were always performed as a single continuous process and without intermediate storage steps.

### Extraction of HIV-1 genomes from NMIA-modified particles.

RNA genomes subjected to reaction with NMIA in virio were gently extracted from viral particles as described [37]. In sum, concentrated samples of virus particles (in 550 μl of NMIA reaction buffer) were incubated at 22 °C with 5 μl of proteinase K (20 mg/ml), 33.5 μl of 1 M Tris-HCl (pH 7.5), 13.4 μl of 5 M NaCl, 1.34 μl of 0.5 M EDTA, 6.7 μl of 1 M DTT, and 4 μl of glycogen (20 mg/ml) for 30 min. RNA was purified by three consecutive extractions with phenol:chloroform:isoamyl alcohol (25:24:1), followed by precipitation with ethanol. Samples were resuspended in 1/2× TE to a concentration of 0.5 μM, based on quantitative RT-PCR analysis.

### Extraction and SHAPE analysis of HIV-1 genomes from native particles.

For the ex virio state, pelleted viral particles were dissolved in 1 ml of 50 mM Hepes (pH 8.0), 0.5 mM EDTA, 200 mM NaCl, 1% (w/v) SDS, and 100 μg/ml proteinase K and digested for 30 min at 22 °C. The RNA was then extracted against phenol-chloroform, and the resulting deproteinized genomes were then aliquoted (2 pmol) and flash frozen at −80 °C. For SHAPE analysis, the ex virio RNA was treated with NMIA using the same procedure as for modification of the in vitro RNA (described below), except that the initial 90 °C heat step was omitted, and the time for incubation in folding buffer was reduced to 10 min.

### RNA transcript.

A DNA template encoding the 5′ 976 nts of the HIV-1 genome and containing a promoter for T7 RNA polymerase was generated by PCR (2 ml; 20 mM Tris [pH 8.4], 50 mM KCl, 2.5 mM MgCl_2_, 0.5 μM forward [5′-TAATA CGACT CACTA TAGGT CTCTC TGGTT AGACC] and reverse [5′-CTATC CCATT CTGCA GCTTC C] primers, approximately 1 μg of plasmid template containing a partial sequence of the HIV-1 pNL4–3 molecular clone [obtained from the National Institutes of Health AIDS Research and Reference Reagent Program], 200 μM each dNTP, and 25 units Taq polymerase; 34 cycles). The PCR product was recovered by ethanol precipitation and resuspended in 300 μl of TE (10 mM Tris [pH 8], 1 mM EDTA). Transcription reactions (3 ml; 37 °C; 5 h; 40 mM Tris [pH 8.0], 5 mM MgCl_2_, 10 mM DTT, 4 mM spermidine, 0.01% Triton X-100, 4% [w/v] PEG 8000, 300 μl of PCR product, and 2 mM each NTP) were initiated by adding 300 μl of 1 mg/ml T7 RNA polymerase [62]. The RNA product was precipitated and purified by denaturing polyacrylamide gel electrophoresis (5% acrylamide, 1× TBE 7 M urea), excised from the gel, and recovered by electroelution. The purified RNA (0.6 nmol) was resuspended in 100 μl of TE.

### Nondenaturing gel electrophoresis of transcript RNA.

RNA transcript (0.54 pmol, 3 μl, 1/2× TE) was combined with internally [^32^P]-labeled transcript RNA (7 fmol, 1 μl) and denatured by heating at 95 °C for 5 min and placing on ice. Folding buffer (1 μl of 5×; 250 mM Hepes [pH 8], 1 M potassium acetate [pH 8], 25 mM MgCl_2_) was added, and the sample was incubated at 37 °C for 1 h. Dimer markers were generated by the same procedure, except that 5 pmol of RNA were used and incubation was at 60 °C; monomer markers were generating by heating labeled transcript RNA (7 fmol, 5 μl) to 95 °C for 10 min in 1/2× TE. Gel loading dye (30% glycerol, 1× TBE, 7 mM MgCl_2_, 0.01% xylene cyanol; 1.67 μl) was added, and all samples were incubated on ice until resolution by electrophoresis (2 μl of sample per well, 4% polyacrylamide, 1× TBE, 7 W, 2.5 h) either in the absence or presence of 7 mM MgCl_2_. If MgCl_2_ was present during the separation, it was added to both the running buffer and the gel; the running buffer was recycled every 10 min to maintain a constant [Mg^2+^] in the gel.

### Modification of transcript RNA.

RNA (2 pmol) in 14.4 μl of 1/2× TE was refolded by heating at 95 °C, placing on ice, adding 3.6 μl of folding buffer, and incubating at 37 °C for 60 min. The folded RNA was divided equally between two tubes and treated with either NMIA [22,23] (1 μl, 32 mM in DMSO) or neat DMSO (1 μl) and allowed to react for 60 min at 37 °C. RNA from the (+) and (−) NMIA reagent experiments was recovered by ethanol precipitation and resuspended in 10 μl of TE.

### Detection of 2′-*O*-adducts by primer extension.

In vitro transcript or authentic genomic RNA (1 pmol, 10 μl, in 1× TE) corresponding to either the (+) or (−) NMIA reactions was heated to 95 °C for 3 min and cooled on ice for 1 min. Fluorescently labeled primer (3 μl) was added to the (+) (0.2 μM Cy5) and (−) (0.4 μM WellRED D3) NMIA reactions, respectively, and primer-template solutions were incubated at 65 °C for 5 min and 35 °C for 10 min. Primer extension was initiated by addition of enzyme mix (6 μl; 250 mM KCl; 167 mM Tris-HCl [pH 8.3]; 1.67 mM each dATP, dCTP, dITP, and dTTP; 10 mM MgCl_2_; 52 °C, 1 min) and Superscript III (1 μl, 200 units; Invitrogen). Extension continued at 52 °C for 15 min. Sequencing reactions used to identify peaks in the (+) and (−) reagent experiments contained transcript RNA (1 pmol, in 9 μl of TE), 3 μl of primer (2 μM WellRED D2 or 1.2 μM LICOR IR 800), enzyme mix (6 μl), ddNTP solution (1 μl; 0.25 mM ddGTP and 10 mM other nucleotides), and Superscript III (1 μl). Four sets of primers were used that were complementary to positions 342–363 (5′-CGCTT AATAC CGACG CTCTC GC), 535–555 (5′-CTTCT GATCC TGTCT GAAGG G), 743–762 (5′-CCATT TGCCC CTGGA GGTT C), or 956–976 (CTATC CCATT CTGCA GCTTC C). Depending on the quality of synthesis, primers were purified by denaturing gel electrophoresis (20% polyacrylamide, 1× TBE, 7 M urea; dimensions 0.75 cm × 28.5 cm [w] × 23 cm [h]; 32W; 90 min) and passively eluted into 1/2× TE overnight. The four reactions corresponding to a complete hSHAPE analysis ((+) NMIA, (−) NMIA, and two sequencing reactions) were combined and precipitated with ethanol in the presence of acetate, EDTA, and glycogen. Pellets were washed twice with 70% ethanol, dried under vacuum, and resuspended in deionized formamide. cDNA samples were separated on a 33-cm × 75-μm capillary using a Beckman CEQ 2000XL DNA sequencer.

### Data processing.

Raw fluorescence intensity versus elution time profiles were analyzed using a draft software suite called ShapeFinder. ShapeFinder is derived from BaseFinder [25], which is a modular, extensible software package originally designed for DNA base calling and sequence analysis, and is currently being refined for analysis of quantitative hSHAPE reactivity information (S. M. Vasa, N. Guex, K. A. Wilkinson, K. M. Weeks and M. C. Giddings, unpublished data). For readers interested in immediate access to this software, a beta version is available for download at http://rd.plos.org/pbio.0060096.1. Processing steps included (1) baseline correction, (2) color separation to correct for spectral overlap of the fluorescent dyes, and (3) mobility shift correction to align corresponding peaks in the four channels. Areas under each peak in the (+) and (−) NMIA traces were obtained by (1) peak detection and interpolation to align peaks in each channel with the RNA sequence and (2) performing a whole-trace Gaussian-fit integration. Integrated peak intensities were corrected for signal decay [63] as a function of read length by assuming a constant probability for extension at each nucleotide position, after excluding the 2% of most-highly reactive peaks:

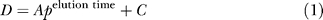



Where *D* is the signal decay adjustment factor, *A* and *C* are scaling factors that reflect the arbitrary initial and final intensities of the trace, and *p* is the probability of extension at each nucleotide. Typical values for *p* spanned 0.995–0.999 for elution times in units of 2 Hz. Each peak intensity calculated at the same elution time was divided by *D*. SHAPE intensities at each individual nucleotide were examined manually to identify positions where high background was present in the (−) reagent control experiment. A very small number of positions fell into this category (<6%) and were marked as containing no data. Quantitative SHAPE reactivities for individual datasets were normalized to a scale in which 0 indicates an unreactive site and the average intensity at highly reactive sites is set to 1.0. The normalization factor for each dataset was determined by first excluding the most-reactive 2% of peak intensities and then calculating the average for the next 8% of peak intensities. All reactivities were then divided by this average. This simple normalization procedure places all absolute reactivities on a scale of 0 to approximately 1.5 (ordinate, Figure 2D). Normalized hSHAPE reactivities from each primer extension reaction were processed independently. Processed traces were then found to fall on the same absolute scale, without further adjustment (for example, compare overlap of closed and open columns, Figure 3A). For each state, SHAPE information was obtained by combining information from four overlapping reads of approximately 300 nts each; two to three independent repetitions were obtained for each read; and standard deviations were small, on average 0.1 normalized SHAPE unit or less.

### Ability of hSHAPE to detect small changes in RNA structure.

These experiments are outlined in Figure S1. PCR templates for the 362-mer transcript and U^22^CU^24^-deletion mutants were produced in the context of a 3′ structure cassette [22] using the same reverse cassette primer (5′-GAACC GGACC GAAGC CCGAT TGGTA ACCCG AAGGT CACTT CCGCT TAATA CTGAC GCTC) and different forward primers (5′-TAATA CGACT CACTA TAGGC CTTCG GGCCA AGGTC TCTCT GGTTA GACC) and (5′-TAATA CGACT CACTA TAGGC CTTCG GGCCA AGGTC TCTCT GGTTA GACCA GAGAG CCTGG G), respectively. tRNA(lys3) was dissociated from the ex virio RNA by heating to 90 °C for 3 min and refolding 10 min in reaction buffer at 37 °C.

### Incorporation of hSHAPE constraints into RNAstructure.

SHAPE intensities were converted into a pseudo-free energy change term in the RNAstructure program [28] (K. E. Deigan, T. W. Li, D. H. Mathews, and K. M. Weeks, unpublished data) using:

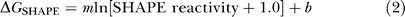
which is applied to each nucleotide in each stack of two base pairs. Therefore, the pseudo-free energy is added twice per nucleotide paired in the interior of a helix and once per nucleotide paired at the end of a helix. The intercept, *b*, is the free-energy bonus for formation of a base pair with zero or low SHAPE reactivity, whereas *m*, the slope, drives an increasing penalty for base pairing as the SHAPE reactivity increases. The *b* and *m* parameters were −0.6 and 1.7 kcal/mol, respectively (per nucleotide). The maximum allowed distance between paired bases was restrained to be 300 nts or less. Increasing the maximum pairing distance to 600 nts yielded a series of short, poorly predicted, and transient pairings. The reactivity of the nucleotides participating in these interactions could be explained by shorter distance pairings. To determine the pairing persistence, structures were also computed for larger values of the *b* and *m* parameters, which has the effect of increasing the contribution of the SHAPE reactivity information on the secondary structure calculation. Helices considered to be highly predicted persisted even when these parameters were set to values as high as *b* = 0 and *m* > 4. RNAstructure is available for download at http://rd.plos.org/pbio.0060096.2.

### Statistical analyses, general procedures.

To analyze differences in SHAPE reactivities between groups of nucleotides, such as comparing the 5′ regulatory versus 3′ protein coding regions, several tests were applied to determine the statistical characteristics of the subgroups and to determine the appropriate statistical measures of differences. We applied Levene's test to analyze homoscedasticity, which confirmed that the SHAPE reactivity variances between analyzed groups of nucleotides are equivalent. However, quantile-quantile (Q-Q) plots of these data indicated departures from normality for the SHAPE reactivity data, so the standard Student *t*-test could not be applied. Instead, we applied the Wilcoxon rank sum test to analyze the statistical SHAPE reactivity differences between groups, which eliminates dependence on normality by rank-ordering the reactivity data and deriving statistics from the rankings. In the small number of cases in which reactivity data were missing for a nucleotide in one group, then that nucleotide was removed from the other groups in a given comparison. All analyses were performed using the open source R package (version 2.6.1) [64].

*Differences in structure between regulatory and coding domains.* SHAPE reactivity data for the ex virio state were divided into two groups corresponding to the 5′ regulatory (nucleotides 1–335) and Gag coding sequences (nucleotides 336–906) and analyzed by the Wilcoxon rank sum test. Differences between the 5′ regulatory and 3′ coding regions were highly significant (*p* < 0.0001).

*Absence of differences between TAR and DIS SHAPE reactivities for in vitro transcript, ex virio, and AT-2–treated states.* Reactivity data were analyzed for the TAR (nucleotides 1–57) and DIS (nucleotides 236–282) regions. Three groups of reactivities were collected, reflecting each RNA state (in vitro, ex virio, and AT-2 treated) with 141 and 129 nts for the TAR and DIS regions, respectively. We applied a one-way analysis of variance (ANOVA) to the three groups for each of the regions, to test whether they displayed statistically significant differences among groups for either region. The F ratio was calculated to determine whether the means of individual groups, corresponding to the three states, represent significant variation from the overall mean. The initial F ratio values calculated showed no significant difference between groups, with *p*-values of 0.63 for TAR and 0.98 for DIS, respectively. However, because SHAPE reactivities are not normally distributed, the F ratio can be inaccurate. To assess whether the initial F ratio was representative of the underlying distribution, we performed bootstrap resampling of the SHAPE data from the groups, with replacement [65]. We recalculated the F ratio for each of 15,000 repetitions, then calculated *p*-values by determining the number of times F ratios with larger (more extreme) values were obtained. The bootstrap–re-estimated *p*-values were 0.625 for TAR and 0.974 for DIS, in close agreement with the initial calculation. This indicates that the original F ratio, which showed no statistical difference between these groups, is representative of the lack of difference between the TAR and DIS structures in these three HIV-1 RNA genome states.

*Statistical significance of nucleocapsid binding sites.* SHAPE reactivity data were divided into two groups corresponding to the in virio and AT-2–treated states. For each state, the nucleotides were divided into the three nucleocapsid protein binding classes (Figures 7 and 8). This resulted in two groups of 34, 42, and 28 nts for the Class 1, 2, and 3 sites, respectively. Each of these were compared using the Wilcoxon rank sum test. For all three classes of nucleocapsid protein interaction sites, *p*-values < 0.001, indicating statistically significant differences in the SHAPE reactivity between in virio and AT-2–treated states. We also performed the inverse analysis, using all nucleotides that were not associated with any of the three classes of nucleocapsid sites. The Wilcoxon test yielded a *p*-value of 0.859, indicating that nucleotides outside of these sites were statistically equivalent.

Information content of nucleocapsid protein binding sites was calculated as described [54,55]. Because the number of sequences was relatively small, information content shows some variation depending on the specific alignment. For example, information content was 9.6 bits for the alignment shown in Figure 10; alternative alignments yielded information contents of 8 to 12 bits.

## Supporting Information

Dataset S1 SHAPE Reactivity Data This dataset contains the complete SHAPE reactivity data for the ex virio, in virio, AT-2 treated, and in vitro transcript states.
(346 KB XLS)

Figure S1 hSHAPE Accurately Detects Small Changes in HIV-1 Genome Structure In this figure, structures are illustrated schematically at the left. Representative nucleotides are colored by their experimental SHAPE reactivity using the scale shown in Figure 4. Reactivity histograms are shown at the right; large upward or downward pointing colored arrows indicate increases or decreases in SHAPE reactivity, respectively.
(A) Deletion of the U^22^CU^24^ bulge in TAR. Native and ΔU^22^CU^24^ histograms are black and purple, respectively.
(B) SHAPE analysis of the 79–85/443–339 pseudoknot. Pseudoknot structure was analyzed for the 976 nt in vitro transcript (black histogram) as compared to a 362 nt RNA that lacks sequences required to form the pseudoknot (cyan).
(C) Ex virio genomic RNA bound to tRNA(lys) (red) undergoes a local rearrangement to maximize intra-molecular pairs (orange) upon heating and refolding.
(294 KB PDF)

### Accession Numbers

The GenBank (http://www.ncbi.nlm.nih.gov/Genbank) accession number for the pNL4–3 molecular clone is AF324493.

## Abbreviations

AT-2: Aldrithiol-2; hSHAPE; high-throughput selective 2′-hydroxyl acylation analyzed by primer extension
nt: nucleotide
SD: splice-donor
SHAPE: selective 2′-hydroxyl acylation analyzed by primer extension.

## References

[pbio-0060096-b001] Coffin JM, Hughes SH, Varmus HE (1997). Retroviruses.

[pbio-0060096-b002] Frankel AD, Young JAT (1998). HIV-1: fifteen proteins and an RNA. Annu Rev Biochem.

[pbio-0060096-b003] Berkowitz R, Fisher J, Goff SP (1996). RNA packaging. Curr Top Microbiol Immunol.

[pbio-0060096-b004] Rein A (2004). Take two. Nature Struct Mol Biol.

[pbio-0060096-b005] D'Souza V, Summers MF (2005). How retroviruses select their genomes. Nat Rev Microbiol.

[pbio-0060096-b006] Rein A, Henderson LE, Levin JG (1998). Nucleic-acid-chaperone activity of retroviral nucleocapsid proteins: significance for viral replication. Trends Biochem Sci.

[pbio-0060096-b007] Levin JG, Guo J, Rouzina I, Musier-Forsyth K (2005). Nucleic acid chaperone activity of HIV-1 nucleocapsid protein: critical role in reverse transcription and molecular mechanism. Prog Nucleic Acid Res Mol Biol.

[pbio-0060096-b008] Baudin F, Marquet R, Isel C, Darlix J-L, Ehresmann B (1993). Functional sites in the 5′ region of human immunodeficiency virus type 1 form defined structural domains. J Mol Biol.

[pbio-0060096-b009] Berkhout B (1996). Structure and function of the human immunodeficiency virus leader RNA. Prog Nucleic Acid Res Mol Biol.

[pbio-0060096-b010] Harrison GP, Miele G, Hunter E, Lever AM (1998). Functional analysis of the core human immunodeficiency virus type 1 packaging signal in a permissive cell line. J Virol.

[pbio-0060096-b011] Clever JL, Sassetti C, Parslow TG (1995). RNA secondary structure and binding sites for gag gene products in the 5′ packaging signal of human immunodeficiency virus type 1. J Virol.

[pbio-0060096-b012] Clever JL, Mirandar D, Parslow TG (2002). RNA structure and packaging signals in the 5′ leader region of the human immunodeficiency virus type 1 genome. J Virol.

[pbio-0060096-b013] Berkhout B, Ooms M, Beerens N, Huthoff H, Southern E (2002). In vitro evidence that the untranslated leader of the HIV-1 genome is an RNA checkpoint that regulates multiple functions through conformational changes. J Biol Chem.

[pbio-0060096-b014] Paillart JC, Skripkin E, Ehresmann B, Ehresmann C, Marquet R (2002). In vitro evidence for a long range pseudoknot in the 5′-untranslated and matrix coding regions of the HIV-1 genomic RNA. J Biol Chem.

[pbio-0060096-b015] Damgaard CK, Andersen ES, Knudsen B, Gorodkin J, Kjems J (2004). RNA interactions in the 5′ region of the HIV-1 genome. J Mol Biol.

[pbio-0060096-b016] Paillart JC, Dettenhofer M, Yu XF, Ehresmann C, Ehresmann B (2004). First snapshots of the HIV-1 RNA structure in infected cells and in virions. J Biol Chem.

[pbio-0060096-b017] Kasprzak W, Bindewald E, Shapiro BA (2005). Structural polymorphism of the HIV-1 leader region explored by computational methods. Nucleic Acids Res.

[pbio-0060096-b018] Michel F, Westhof E (1990). Modelling of the three-dimensional architecture of group I catalytic introns based on comparative sequence analysis. J Mol Biol.

[pbio-0060096-b019] Gutell RR, Lee JC, Cannone JJ (2002). The accuracy of ribosomal RNA comparative structure models. Curr Opin Struct Biol.

[pbio-0060096-b020] Ehresmann C, Baudin F, Mougel M, Romby P, Ebel JP (1987). Probing the structure of RNAs in solution. Nucl Acids Res.

[pbio-0060096-b021] Knapp G (1989). Enzymatic approaches to probing of RNA secondary and tertiary structure. Methods Enzymol.

[pbio-0060096-b022] Merino EJ, Wilkinson KA, Coughlan JL, Weeks KM (2005). RNA structure analysis at single nucleotide resolution by selective 2′-hydroxyl acylation and primer extension (SHAPE). J Am Chem Soc.

[pbio-0060096-b023] Wilkinson KA, Merino EJ, Weeks KM (2005). RNA SHAPE chemistry reveals non-hierarchical interactions dominate equilibrium structural transitions in tRNA^Asp^ transcripts. J Am Chem Soc.

[pbio-0060096-b024] Wilkinson KA, Merino EJ, Weeks KM (2006). Selective 2′-hydroxyl acylation analyzed by primer extension (SHAPE): Quantitative RNA structure analysis at single nucleotide resolution. Nature Protocols.

[pbio-0060096-b025] Giddings MC, Severin J, Westphall M, Wu J, Smith LM (1998). A software system for data analysis in automated DNA sequencing. Genome Res.

[pbio-0060096-b026] Puglisi JD, Tan R, Calnan BJ, Frankel AD, Williamson JR (1992). Conformation of the TAR RNA-arginine complex by NMR spectroscopy. Science.

[pbio-0060096-b027] Mathews DH, Sabina J, Zuker M, Turner DH (1999). Expanded sequence dependence of thermodynamic parameters improves prediction of RNA secondary structure. J Mol Biol.

[pbio-0060096-b028] Mathews DH, Disney MD, Childs JL, Schroeder SJ, Zuker M (2004). Incorporating chemical modification constraints into a dynamic programming algorithm for prediction of RNA secondary structure. Proc Natl Acad Sci USA.

[pbio-0060096-b029] Badorrek CS, Weeks KM (2005). RNA flexibility in the dimerization domain of a gamma retrovirus. Nature Chem Biol.

[pbio-0060096-b030] Mortimer SA, Weeks KM (2007). A fast acting reagent for accurate analysis of RNA secondary and tertiary structure by SHAPE chemistry. J Am Chem Soc.

[pbio-0060096-b031] Workman C, Krogh A (1999). No evidence that mRNAs have lower folding free energies than random sequences with the same dinucleotide distribution. Nucleic Acids Res.

[pbio-0060096-b032] Rivas E, Eddy SR (2000). Secondary structure alone is generally not statistically significant for the detection of noncoding RNAs. Bioinformatics.

[pbio-0060096-b033] Gherghe C, Weeks KM (2006). The SL1-SL2 (stem-loop) domain is the primary determinant for stability of the gamma retroviral genomic RNA dimer. J Biol Chem.

[pbio-0060096-b034] Chen Y, Fender J, Legassie JD, Jarstfer MB, Bryan TM (2006). Structure of stem-loop IV of Tetrahymena telomerase RNA. EMBO J.

[pbio-0060096-b035] Vicens Q, Gooding AR, Laederach A, Cech TR (2007). Local RNA structural changes induced by crystallization are revealed by SHAPE. RNA.

[pbio-0060096-b036] Dann CE, Wakeman CA, Sieling CL, Baker SC, Irnov I (2007). Structure and mechanism of a metal-sensing regulatory RNA. Cell.

[pbio-0060096-b037] Fu W, Gorelick RJ, Rein A (1994). Characterization of human immunodeficiency virus type 1 dimeric RNA from wild-type and protease-defective virions. J Virol.

[pbio-0060096-b038] Sakuragi J, Iwamoto A, Shioda T (2002). Dissociation of genome dimerization from packaging functions and virion maturation of human immunodeficiency virus type 1. J Virol.

[pbio-0060096-b039] Hu WS, Temin HM (1990). Genetic consequences of packaging two RNA genomes in one retroviral particle: pseudodiploidy and high rate of genetic recombination. Proc Natl Acad Sci U S A.

[pbio-0060096-b040] Andersen ES, Jeeninga RE, Damgaard CK, Berkhout B, Kjems J (2003). Dimerization and template switching in the 5′ untranslated region between various subtypes of human immunodeficiency virus type 1. J Virol.

[pbio-0060096-b041] Negroni M, Buc H (2001). Mechanisms of retroviral recombination. Annu Rev Genet.

[pbio-0060096-b042] Badorrek CS, Gherghe CM, Weeks KM (2006). Structure of an RNA switch that enforces stringent retroviral genomic RNA dimerization. Proc Natl Acad Sci U S A.

[pbio-0060096-b043] Paillart JC, Shehu-Xhilaga M, Marquet R, Mak J (2004). Dimerization of retroviral RNA genomes: an inseparable pair. Nature Rev Microbiol.

[pbio-0060096-b044] Das AT, Harwig A, Vrolijk MM, Berkhout B (2007). The TAR hairpin of human immunodeficiency virus type 1 can be deleted when not required for Tat-mediated activation of transcription. J Virol.

[pbio-0060096-b045] Moore MD, Fu W, Nikolaitchik O, Chen J, Ptak RG (2007). Dimer initiation signal of human immunodeficiency virus type 1: its role in partner selection during RNA copackaging and its effects on recombination. J Virol.

[pbio-0060096-b046] Beerens N, Groot F, Berkhout B (2001). Initiation of HIV-1 reverse transcription is regulated by a primer activation signal. J Biol Chem.

[pbio-0060096-b047] Isel C, Westhof E, Massire C, Le Grice SFJ, Ehresmann B (1999). Structural basis for the specificity of the initiation of HIV-1 reverse transcription. EMBO J.

[pbio-0060096-b048] Rice WG, Supko JG, Malspeis L, Buckheit RW, Clanton D (1995). Inhibitors of HIV nucleocapsid protein zinc fingers as candidates for the treatment of AIDS. Science.

[pbio-0060096-b049] Chertova E, Crise BJ, Morcock DR, Bess JW, Henderson LE (2003). Sites, mechanism of action and lack of reversibility of primate lentivirus inactivation by preferential covalent modification of virion internal proteins. Curr Mol Med.

[pbio-0060096-b050] Rossio JL, Esser MT, Suryanarayana K, Schneider DK, Bess JW (1998). Inactivation of human immunodeficiency virus type 1 infectivity with preservation of conformational and functional integrity of virion surface proteins. J Virol.

[pbio-0060096-b051] Dowell RD, Eddy SR (2004). Evaluation of several lightweight stochastic context-free grammars for RNA secondary structure prediction. BMC Bioinformatics.

[pbio-0060096-b052] Mathews DH, Turner DH (2006). Prediction of RNA secondary structure by free energy minimization. Curr Opin Struct Biol.

[pbio-0060096-b053] Doshi KJ, Cannone JJ, Cobaugh CW, Gutell RR (2004). Evaluation of the suitability of free-energy minimization using nearest-neighbor energy parameters for RNA secondary structure prediction. BMC Bioinformatics.

[pbio-0060096-b054] Schneider TD, Stephens RM (1990). Sequence logos: a new way to display consensus sequences. Nucl Acids Res.

[pbio-0060096-b055] Gorodkin J, Heyer LJ, Brunak S, Stormo GD (1997). Displaying the information contents of structural RNA alignments: the structure logos. Comput Appl Biosci.

[pbio-0060096-b056] Fisher RJ, Rein A, Fivash M, Urbaneja MA, Casas-Finet JR (1998). Sequence-specific binding of human immunodeficiency virus type 1 nucleocapsid protein to short oligonucleotides. J Virol.

[pbio-0060096-b057] Vuilleumier C, Bombarda E, Morellet N, Gerard D, Roques BP (1999). Nucleic acid sequence discrimination by the HIV-1 nucleocapsid protein NCp7: a fluorescence study. Biochemistry.

[pbio-0060096-b058] De Guzman RN, Wu ZR, Stalling CC, Pappalardo L, Borer PN (1998). Structure of the HIV-1 nucleocapsid protein bound to the SL3 psi-RNA recognition element. Science.

[pbio-0060096-b059] Amarasinghe GK, De Guzman RN, Turner RB, Chancellor KJ, Wu ZR (2000). NMR structure of the HIV-1 nucleocapsid protein bound to stem-loop SL2 of the psi-RNA packaging signal. Implications for genome recognition. J Mol Biol.

[pbio-0060096-b060] Burns JC, Friedmann T, Driever W, Burrascano M, Yee JK (1993). Vesicular stomatitis virus G glycoprotein pseudotyped retroviral vectors: concentration to very high titer and efficient gene transfer into mammalian and nonmammalian cells. Proc Natl Acad Sci U S A.

[pbio-0060096-b061] Thomas JA, Gagliardi TD, Alvord WG, Lubomirski M, Bosche WJ (2006). Human immunodeficiency virus type 1 nucleocapsid zinc-finger mutations cause defects in reverse transcription and integration. Virology.

[pbio-0060096-b062] Milligan JF, Uhlenbeck OC (1989). Synthesis of small RNAs using T7 RNA polymerase. Methods Enzymol.

[pbio-0060096-b063] Badorrek CS, Weeks KM (2006). Architecture of a gamma retroviral genomic RNA dimer. Biochemistry.

[pbio-0060096-b064] R Development Core Team (2007). R: A language and environment for statistical computing.

[pbio-0060096-b065] Higgins JJ (2003). An introduction to modern nonparametric statistics.

