# Supplementary figures and images for: High-Throughput SHAPE Analysis Reveals Structures in HIV-1 Genomic RNA Strongly Conserved across Distinct Biological States
Source: PLoS Biol. 2008 Apr 29;6(4):e96. doi: 10.1371/journal.pbio.0060096 (PMC2689691; doi:10.1371/journal.pbio.0060096)

A

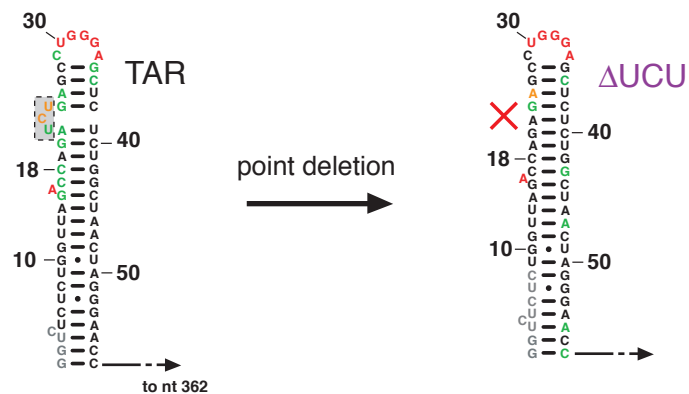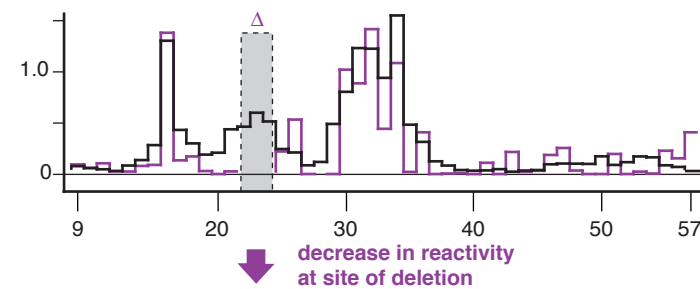

B

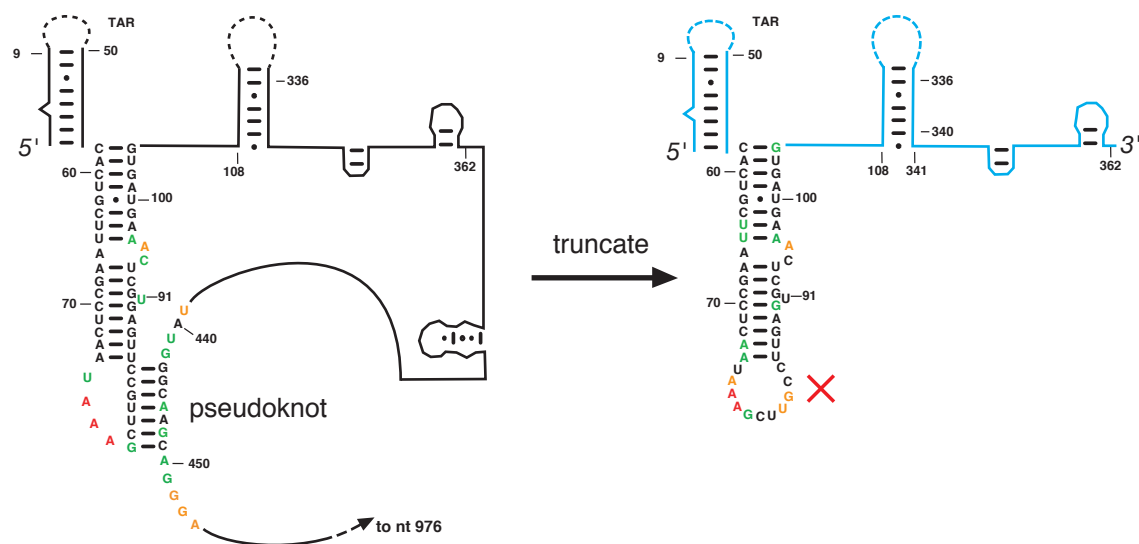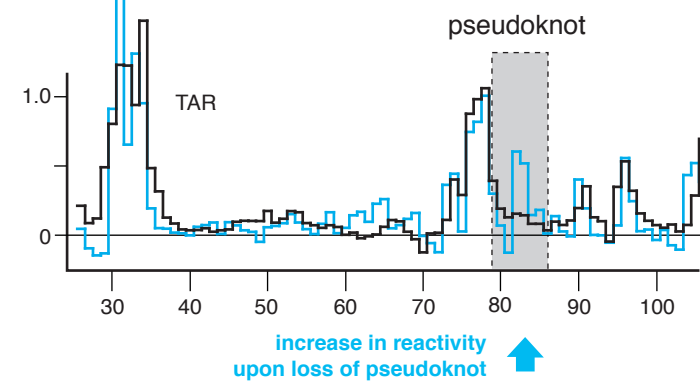

C

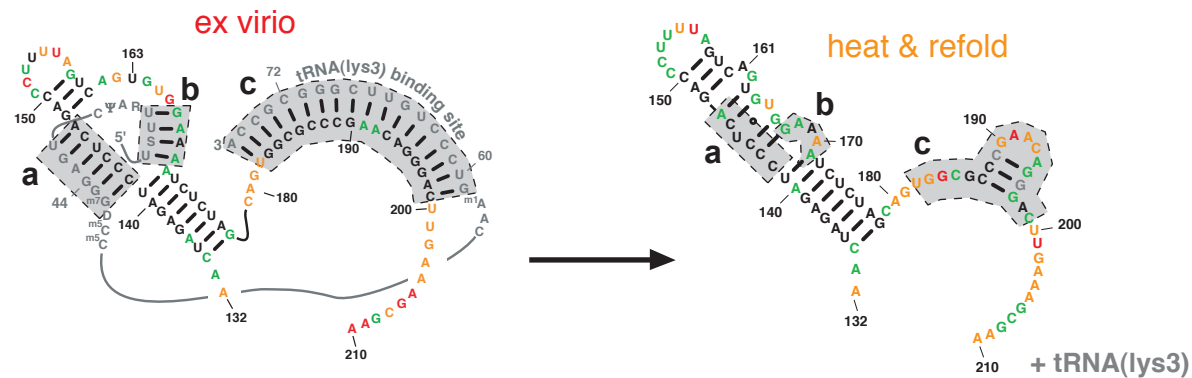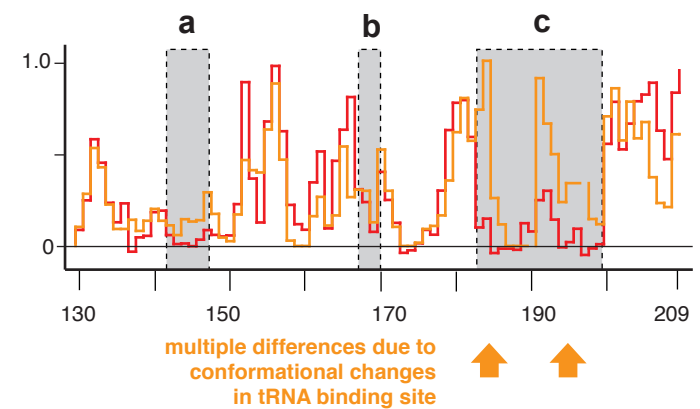

Supplement: Figure S1 — In this figure, structures are illustrated schematically at the left. Representative nucleotides are colored by their experimental SHAPE reactivity using the scale shown in Figure 4. Reactivity histograms are shown at the right; large upward or downward pointing colored arrows indicate increases or decreases in SHAPE reactivity, respectively. (A) Deletion of the U22CU24 bulge in TAR. Native and ΔU22CU24 histograms are black and purple, respectively. (B) SHAPE analysis of the 79–85/443–339 pseudoknot. Pseudoknot structure was analyzed for the 976 nt in vitro transcript (black histogram) as compared to a 362 nt RNA that lacks sequences required to form the pseudoknot (cyan). (C) Ex virio genomic RNA bound to tRNA(lys) (red) undergoes a local rearrangement to maximize intra-molecular pairs (orange) upon heating and refolding. (294 KB PDF) [file pbio.0060096.sg001.pdf]
